# Effect of a High Fat Diet vs. High Carbohydrate Diets With Different Glycemic Indices on Metabolic Parameters in Male Endurance Athletes: A Pilot Trial

**DOI:** 10.3389/fnut.2022.802374

**Published:** 2022-04-11

**Authors:** Denise Zdzieblik, Hilke Friesenborg, Albert Gollhofer, Daniel König

**Affiliations:** ^1^Department for Nutrition, Institute for Sports and Sports Science, University of Freiburg, Freiburg, Germany; ^2^Centre for Sports Science and University Sports, Department for Nutrition, Exercise and Health, University of Vienna, Vienna, Austria; ^3^Department for Nutrition, Exercise and Health, Faculty of Life Sciences, University of Vienna, Vienna, Austria

**Keywords:** high fat, high carbohydrate, glycemic index, endurance, lactate, respiratory exchange ratio, fat oxidation, incremental test

## Abstract

Consuming low glycemic carbohydrates leads to an increased muscle fat utilization and preservation of intramuscular glycogen, which is associated with improved flexibility to metabolize either carbohydrates or fats during endurance exercise. The purpose of this trial was to investigate the effect of a 4-week high fat low carbohydrate (HFLC-G: ≥65% high glycemic carbohydrates per day; *n* = 9) vs. high carbohydrate low glycemic (LGI-G: ≥65% low glycemic carbohydrates daily; *n* = 10) or high glycemic (HGI-G: ≥65% fat, ≤ 50 g carbohydrates daily; *n* = 9) diet on fat and carbohydrate metabolism at rest and during exercise in 28 male athletes. Changes in metabolic parameters under resting conditions and during cycle ergometry (submaximal and with incremental workload) from pre- to post-intervention were determined by lactate diagnostics and measurements of the respiratory exchange ratio (RER). Additionally, body composition and perceptual responses to the diets [visual analog scale (VAS)] were measured. A significance level of α = 0.05 was considered. HFLC-G was associated with markedly decreased lactate concentrations during the submaximal (−0.553 ± 0.783 mmol/l, *p* = 0.067) and incremental cycle test [−5.00 ± 5.71 (mmol/l) × min; *p* = 0.030] and reduced RER values at rest (−0.058 ± 0.108; *p* = 0.146) during the submaximal (−0.078 ± 0.046; *p* = 0.001) and incremental cycle test (−1.64 ± 0.700 RER × minutes; *p* < 0.001). In the HFLC-G, fat mass (*p* < 0.001) decreased. In LGI-G lactate, concentrations decreased in the incremental cycle test [−6.56 ± 6.65 (mmol/l) × min; *p* = 0.012]. In the LGI-G, fat mass (*p* < 0.01) and VAS values decreased, indicating improved levels of gastrointestinal conditions and perception of effort during training. The main findings in the HGI-G were increased RER (0.047 ± 0.076; *p* = 0.117) and lactate concentrations (0.170 ± 0.206 mmol/l, *p* = 0.038) at rest. Although the impact on fat oxidation in the LGI-G was not as pronounced as following the HFLC diet, the adaptations in the LGI-G were consistent with an improved metabolic flexibility and additional benefits regarding exercise performance in male athletes.

## Introduction

It has been known for more than 30 years that high carbohydrate diets improve performance during prolonged endurance exercises. Timing, composition, and amount of carbohydrates to optimize endurance capacity have been tested in a large number of scientific investigations (1). Compared to fats and proteins, the utilization of carbohydrates enables a high energy flow rate and an increased energy efficiency in the muscles. During exercise, at ~80–85% of maximum oxygen consumption (VO_2_) in moderately active individuals, carbohydrates are predominantly metabolized (2, 3). However, the carbohydrate stores in the liver and muscles (~1,500–2,000 kcal) are limited (4, 5). In addition to carbohydrates, fats are an important source of energy during physical activity. Free fatty acids originating from the adipocytes and intramuscular triglycerides are used to generate energy. Due to their low energy flow rate, the percentage contribution of fat oxidation to total energy provision is low during short intensive exercises and increases with the duration of the exercise and the decrease in intensity (6).

The intake of high glycemic carbohydrates and the resulting high insulinemic response are one of the strongest inhibitors of fat oxidation (7). Therefore, several clinical studies focused on improving fat utilization during endurance exercise by high fat low carbohydrate diets (8). The concept of a high-fat (>60% of daily energy intake) and low-carbohydrate (<25% of daily energy intake) diet has been discussed since 1985. The current state of evidence suggests a diet with less than 50 g of carbohydrates per day (9). A metabolic adaptation toward increased fat oxidation is usually achieved following a 2- to 4-week dietary intervention (9, 10). However, high fat diets are associated with an impaired carbohydrate utilization during higher intensities (11–14) and, consequently, with small positive effects on endurance performance even with a carbohydrate restoration phase prior to competitions (9). This altered metabolic flexibility can be explained by a reduced enzyme activity in the carbohydrate metabolism due to a reduced training effect within the carbohydrate metabolism following reduced carbohydrate availability or reduced signaling at low glycogen concentrations (2, 3).

In this context, a promising concept might be the consideration of the glycemic index (GI) in the diet. It was shown that the fat oxidation during exercise increased after the consumption of low GI carbohydrates compared to high GI carbohydrates (15–19). This effect can be explained by a reduced postprandial insulin release which leads to an increased plasma concentration of free fatty acids and increased fat oxidation in the skeletal muscles (2, 3). In consequence, intramuscular and intrahepatic glycogen stores are spared and can be used during higher intensities of exercises. In addition to the GI, the glycemic load (GL) which considers the amount of carbohydrates in a given serving of a food also affects blood glucose concentrations and insulin responses (20). To the best of our knowledge, studies comparing high fat with high carbohydrate diets have not considered the GI or GL of the carbohydrates. In addition, long-term investigations examining the influence of the GI or GL are limited and without a high fat low carbohydrate control.

Therefore, the aim of the current pilot study was to examine the impact of a high fat low carbohydrate (HFLC-G) vs. high carbohydrate low glycemic (LGI-G) vs. high carbohydrate high glycemic (HGI-G) diet on metabolic parameters, body composition, and perceptual responses to the diets.

## Materials and Methods

### Study Design and Participants

The study was designed as a monocentric, prospective, open pilot trial conducted at the University of Freiburg, Germany. In total, 30 healthy male endurance athletes as distance runners, cyclists, and athletes performing basic endurance training (e.g., soccer, racket sports) aged between 18 and 50 years were recruited (at least three training sessions per week). The sample size was set on 10 participants per group to obtain information for a power analysis to specify meaningful group differences (21). Professional-level athletes (more than five training sessions per week) were not eligible to participate. Reported health problems during or after physical activity or unstable weight and eating behavior were also defined as exclusion criteria. In addition, contraindications to physical activity according to the American College of Sports Medicine guidelines, such as cardiovascular, metabolic, or renal diseases (22) diagnosed from anamnestic data, led to an exclusion of the screened participants. The examination was approved by the Ethical Committee of the University of Freiburg (ETK: 136-16) and registered in the German Clinical Trials Register (DRKS00015521).

**Figure 1:**
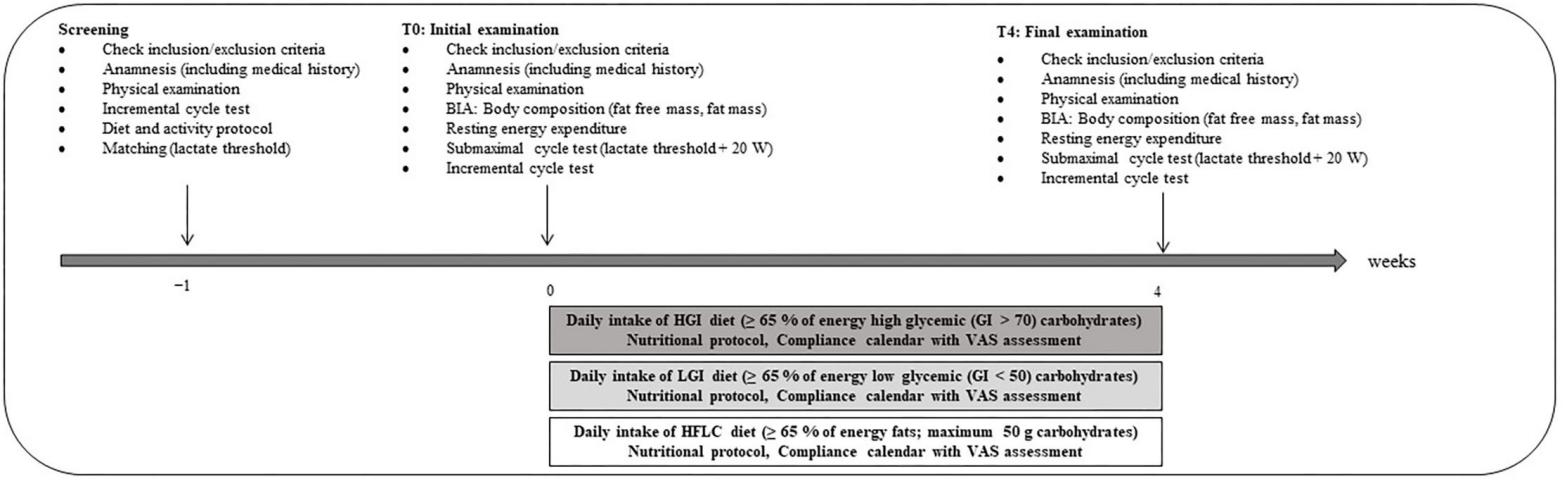
Overview of the study schedule. BIA, bioelectric impedance analysis; VAS, visual analog scale.

For all visits, the participants were advised to arrive at the University of Freiburg in the morning at the same time following a fasted period of 12 h. In addition, general guidelines were to drink 1 L of water in the evening and another 0.5 L in the morning before the measurement to ensure euhydration (23). In addition, the participants had to void bladder before the measurement. Alcoholic beverages had to be avoided 48 h prior to respective examination. All experimental testing was supervised by a licensed physician and experienced researchers. After the start of the intervention, any concerns were clarified directly with the study physician or researcher via telephone call. The study was completed within a timeframe of 4 weeks. The different phases of the study are summarized in Figure 1.

### Screening

Following written informed consent, participants completed a screening with a medical history questionnaire to ensure that the inclusion criteria were met and that there were no risk factors that might be aggravated by the exercise protocols. Furthermore, anthropometric data, including the body composition (as described in the test block below), were measured. An incremental cycling test was performed (cycling 3 min at 100 W, then increasing 20 W per 3 min until exhaustion) on a stationary cycloergometer (Ergoline; ZAN Austria e.U., Steyr-Dietach, Austria) to determine physiological parameters such as VO_2_, respiratory exchange ratio (RER), and submaximal metabolic inflection points (Lactate threshold and individual anaerobic threshold). For this purpose, a breath-by-breath gas analyzer (Innocor® Innovision, Odense, Denmark) was used. Capillary blood samples were collected at rest, every 3 min, and at exhaustion and analyzed using Biosen Glucose and Lactate analyzer (EKF diagnostics GmbH, Barleben/Magdeburg, Germany). Matching for the lactate threshold was used to assign participants to the study groups (HGI-G, LGI-G, and HFLC-G) to minimize baseline differences in the incremental test results. As the first measurable increase in blood lactate concentration during physical activity, the lactate threshold was automatically evaluated by the computer software (Ergonizer 4.7.4, Freiburg, Germany). In addition, participants were asked to complete a 3-day nutrition protocol which included 2 weekdays and 1 day of the weekend before the intervention. The protocols were analyzed with Nutriguide (Nutri-Science GmbH, Pohlheim, Germany). The Freiburg Questionnaire of physical activity was used to protocol frequencies of sports and total time [h/week] of physical and sports activity prior to the intervention (24).

### Test Block

At baseline (T0) and post-intervention (T4), body composition (fat free mass and fat mass) was estimated by using a bioelectric impedance analysis (BIA) with a high reliability for evaluating body composition in healthy adults (25, 26). Participants were assessed on the BIA scale (OMRON BF-500 Medizintechnik GmbH, Mannheim, Germany), which involved entry of the participant's age, height, and male gender. Still wearing the skin-tight clothing, participants stood on the scale barefoot and grasped the handle electrodes for ~10 s until the process was completed. According to the manufacturers recommendation to use this unit in the same environment and daily circumstances, participants were measured in the morning at the same time following a fasted period of 12 h.

The resting energy expenditure (REE) was measured in a quiet, lying position. The actual REE measurement was preceded by a discarded 5-min measuring phase. The respiratory gases were measured for 10 min using the same device from the preliminary incremental cycle test (27). The mean values of VO_2_ and the RER were used in the following Equation (1) by Weir (28) to calculate the REE:


$$\text{REE}\left(\text{kcal/h}\right)\;=\;{\text{VO}}_{\text{2}}\times \left(\text{3}.9+1.\text{1}\times \text{RER}\right)\times \text{60}$$

To determine submaximal exercise metabolism, participants performed a 10-min submaximal cycle test at 20 W above the lactate threshold, which was individually identified in the preliminary incremental cycle test. Blood samples were collected from the ear lobe at rest and at 5 and 10 min. The lactate and glucose concentrations during exercise were determined as the mean value of 5 and 10 min. Values of gas analysis (RER, VO_2_) during exercise were calculated by the mean of each minute.

Ten minutes after completing the submaximal cycle test, an incremental test followed under the same conditions: cycling 3 min at 100 W, then increasing 20 W per 3 min until exhaustion, as described for the screening. For RER, lactate, and glucose values, the area under curve (AUC) was calculated between the start of the test and the final increment completed by all participants before exhaustion (t_n_) to assess the concentrations during the incremental cycle test using the following Equation (2):


$$\begin{array}{l} \text{AU}{\text{C}}_{0-{\text{t}}_{\text{n}}}=\;\frac{1}{2}\overset{20}{\underset{\text{i}=1}{\sum }}\left({\text{t}}_{\text{i}+1}-{\text{t}}_{\text{i}}\right)\times \left({\text{C}}_{\text{i}}-{\text{C}}_{\text{i}+1}\right) \end{array}$$

During the intervention phase, general condition, gastrointestinal condition, and perception of effort (during training session) were evaluated using a visual analog scale (VAS), a validated measurement instrument for quantitative assessment between 0 (“optimal condition”) and 100 (“worst condition”). The distance between “optimal condition” and the point marked by the participant is given in mm by the VAS scale (29).

Participants were instructed to follow the dietary pattern according to their respective group over the time course of 4 weeks:

- HGI-G…≥65% of energy high glycemic (GI > 70) carbohydrates per day
- LGI-G…≥65% of energy low glycemic (GI < 50) carbohydrates per day
- HFLC-G…≥65% of energy fats, maximum 50 g carbohydrates per day

Supplementary Tables S1–S4 summarizes the general nutritional guidelines and example meals for each group. The GI of the foods was based on Foster-Powell et al. (20). All nutritional instructions, including the preparation of the meals, were given by a licensed dietarian who was contacted in case of any questions or concerns about the respective diet. Participants were asked to complete a daily nutrition protocol during the intervention by quantifying the consumed food using household measurements. For self-monitoring the nutritional compliance, participants were instructed to use the diet tracking apps. The protocols were analyzed with Nutriguide (Nutri-Science GmbH, Pohlheim, Germany). The ingestion of ergogenic supplements during or prior to the intervention was defined as exclusion criteria. Changes in physical activity behavior during the intervention led to exclusion of the participants.

### Statistical Analysis

Since the present investigation was conducted as pilot trial, no hierarchy for the efficacy endpoints had been defined in the study protocol. The statistical evaluation was performed to determine an adequate sample size and the primary outcome of a main RCT study which will be designed on the basis of the present study protocol.

All data are presented as mean ± standard deviation (SD). Medians (Md) were additionally presented if outliers were identified by the interquartile range method. SPSS statistics (IBM SPSS Statistics for Windows, Version 25.0. Armonk, NY: IBM Corp.) was used for all statistical analyses. All the tests in the descriptive analysis were performed as two-sided tests, and the significance level was set at α = 0.05.

Data distribution was examined with a Shapiro–Wilk test. If variable data of all groups were normally distributed, the homogeneity of the baseline values between the study groups was checked using one-way ANOVA. In addition, the mean differences obtained from all three groups were compared using one-way ANOVA. The Gabriel *post-hoc* test was performed to identify the groups that differed significantly. The Kruskal–Wallis test was used when data cannot be assumed to be normally distributed. Following a significant Kruskal–Wallis test, pairwise comparisons using the Dunn-Bonferroni approach were automatically produced. The significance of changes from baseline to post-intervention in the respective endpoints within groups were analyzed with the paired sample *t*-test or Wilcoxon signed-rank test. As a magnitude of the change in the respective outcomes, the minimally important difference (MID) was calculated. The value of 0.5 SD can serve as the smallest change from baseline to post-intervention that participants perceive as important (30). Furthermore, the effect sizes were calculated from differences in means between baseline and post-intervention and between groups at the end of the investigation (Cohen's d).

## Results

### Subjects

**Figure 2:**
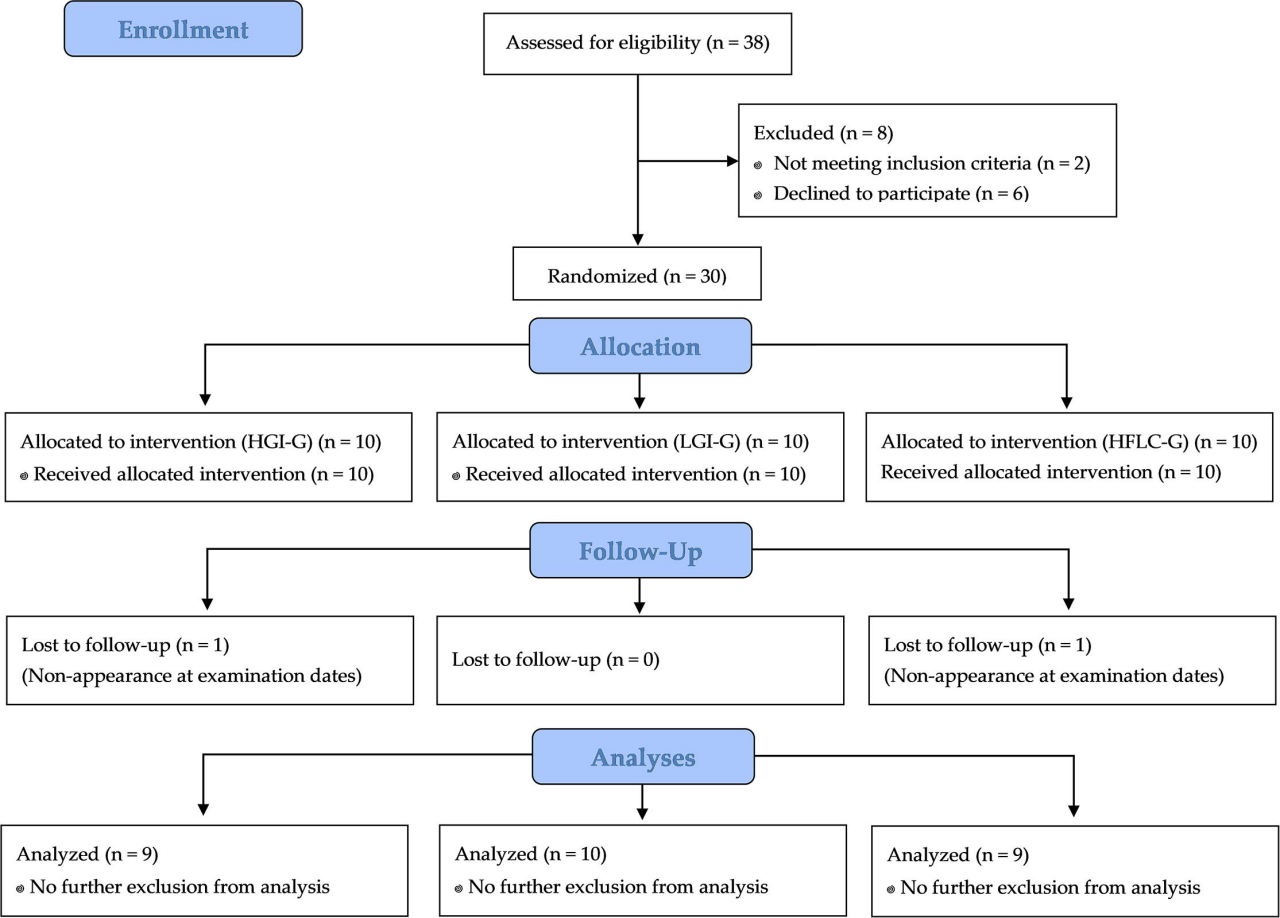
Flow chart of subject recruitment, randomization, and follow up.

A total of 30 men met the inclusion criteria and were allocated to the intervention groups (Figure 2). Twenty-eight participants completed the trial and were included in the statistical analysis. In the HGI-G 9, the LGI-G 10, and the HFLC-G 9, participants were, respectively, analyzed. The participants of the HGI-G were slightly older (27.2 ± 9.00 years) than the participants of the LGI-G (24.2 ± 2.30 years) and HFLC-G (24.8 ± 4.21 years) due to one outlier (50 years). However, the difference was not statistically significant (*p* = 0.489). The mean height was 1.82 ± 0.08 m for HGI-G, 1.77 ± 0.06 m for LGI-G, and 1.79 ± 0.06 m for HFLC-G with no significant group differences (*p* = 0.185). Drop outs were whose who had voluntarily withdrawn from participation after the initial examination. No adverse events were noted, and no pathological findings were observed in the routine anamnesis. The analysis of the initial activity protocols revealed no significant differences (*p* = 0.822) between the HGI-G (7.79 ± 2.65 h/week), LGI-G (7.88 ± 2.83 h/week), and HFLC-G (7.15 ± 2.60 h/week). Endurance running, cycling, team sports, and cross-country skiing were the main reported activities in all groups.

### Nutritional Protocol

As shown in Table 1, no significant baseline differences between the study groups were detected for the nutritional protocols. As a result of the nutritional guidelines in this study, the fat and carbohydrate intake changed statistically significantly (*p* < 0.01) in all study groups. A statistically significant increase in protein intake could be observed in the LGI-G and HFLC-G (*p* < 0.001). Furthermore, the glycemic index of the consumed foods decreased statistically significantly (*p* < 0.001) in the LGI-G and increased statistically significantly (*p* < 0.001) in the HGI-G during the intervention (Table 1). There were no significant differences between the participants' mean energy intake during the course of the study (*p* = 0.887). The carbohydrate and fat intake differed statistically significantly between groups (*p* < 0.001). Moreover, the analysis of the nutritional protocols revealed a statistically significantly lower glycemic index in the LGI-G compared with the HGI-G during the intervention (*p* < 0.001). The daily intake of carbohydrates was higher in the HGI-G than in the LGI-G, with no group differences for the absolute (*p* = 0.979) or percentage (*p* = 0.981) carbohydrate intake. Referring to the body weight, the LGI-G had even a higher carbohydrate intake than the HGI-G, with no statistically significant group difference (*p* = 0.514). The fat intake of the HFLC-G was statistically different from the HGI-G (*p* < 0.001) and the LGI-G (*p* < 0.001). The protein intake also differed significantly between groups (*p* < 0.001). The *post-hoc* analysis revealed a statistically significant higher (*p* < 0.001) protein intake (percent of energy) in the HFLC-G compared with the HGI-G. The average percentage of protein intake of the LGI-G was statistically significantly (*p* = 0.029) lower than in the HFLC-G, with a tendency toward a significance (*p* = 0.057) higher than in the HGI-G. Taking the body weight into account, the protein intake during the intervention was only statistically significantly different between the HGI-G and HFLC-G (*p* < 0.001).

**Table 1:** Dietary patterns at baseline and following the nutritional concepts.

	**HGI-G (*****n*** **= 9)**		**LGI-G (*****n*** **= 10)**		**HFLC-G (*****n*** **= 9)**		***p*-value**
	**T0**	**During**	**T0**	**During**	**T0**	**During**	**ANOVA**
Energy [kcal]	2,392 ± 397	2,386 ± 450	2,367 ± 512	2,377 ± 537	2,524 ± 505	2,498 ± 557	0.855
Fat (g)	96.7 ± 21.8	47.7 ± 12.8^**^	95.8 ± 26.2	44.4 ± 10.2^***^	94.8 ± 21.1	183.2 ± 53.2^***^	**<0.001**
Fat (g/kg BW)	1.28 ± 0.330	0.620 ± 0.118^**^	1.34 ± 0.406	0.626 ± 0.181^***^	1.21 ± 0.255	2.37 ± 0.787^**^	**<0.001**
Fat (% of energy)	37.9 ± 7.77	18.0 ± 3.08^***^	35.9 ± 6.13	17.3 ± 4.92^***^	34.6 ± 6.30	65.1 ± 5.48^***^	**<0.001**
Carbohydrates (g)	282.3 ± 79.4	386.5 ± 83.7^**^	279.9 ± 76.9	373.6 ± 107.9^**^	326.6 ± 90.6	51.3 ± 14.1^***^	**<0.001**
Carbohydrates (g/kg BW)	3.67 ± 0.838	5.09 ± 1.14^**^	4.02 ± 1.57	5.32 ± 2.12^**^	4.22 ± 1.38	0.662 ± 0.198^***^	**<0.001**
Carbohydrates (% of energy)	48.0 ± 8.98	66.2 ± 3.93^**^	46.2 ± 9.30	63.8 ± 5.41^**^	52.4 ± 7.49	9.24 ± 4.83^***^	**<0.001**
Glycemic Index (GI)	53 ± 7	74 ± 3^***^	55 ± 8	39 ± 4^***^			**<0.001**
Protein (g)	86.5 ± 19.9	81.1 ± 15.6	84.4 ± 16.7	105.2 ± 17.9^***^	78.5 ± 16.0	149.1 ± 38.3^***^	**<0.001**
Protein (g/kg BW)	1.16 ± 0.347	1.07 ± 0.215	1.18 ± 0.289	1.48 ± 0.371^***^	1.01 ± 0.208	1.91 ± 0.468^***^	**<0.001**
Protein (% energy)	14.8 ± 2.29	14.0 ± 1.80	14.7 ± 1.64	18.4 ± 1.41^***^	12.8 ± 1.40	24.5 ± 3.16^***^	**<0.001**

*Data represent mean ± SD. P value ANOVA, Differences between groups during intervention with one-way ANOVA*.

** *p < 0.01*;

*** *p < 0.001 within the group from baseline to study intervention. Bold numbers represent statistical significance*.

### Body Composition

The baseline data of the study participants are summarized in Table 2. No significant baseline differences between the study groups were detected in any outcome of the study. The current investigation identified a statistically significant decrease in weight, BMI, and fat mass in the LGI-G and HFLC-G. As a consequence, the percentage of fat free mass increased statistically significantly in the LGI-G and HFLC-G (Table 2). These results were confirmed by the MID and medium effect size in the LGI-G and HFLC-G.

**Table 2:** Body composition and metabolic outcomes at baseline and following the nutritional concepts.

	**HGI-G (*****n*** **= 9)**			**LGI-G (*****n*** **= 10)**			**HFLC-G (*****n*** **= 9)**			***p*-value**
	**T0**	**T4**	***d*_cohen_**	**T0**	**T4**	***d*_cohen_**	**T0**	**T4**	***d*_cohen_**	**ANOVA**
**Body composition and resting metabolism**										
Weight [kg]	76.6 ± 11.0	76.5 ± 11.7	0.009	72.6 ± 11.2	70.2 ± 10.6^**^	0.220	78.4 ± 7.55	75.4 ± 6.41^**^	0.428	**0.001**
BMI [kg/m^2^]	22.9 ± 1.56	22.9 ± 1.83	0.000	23.2 ± 2.53	22.5 ± 2.39^**^	0.284	24.5 ± 1.88	23.6 ± 1.49^**^	0.531	**0.002**
Fat free mass [kg]	63.7 ± 7.54	63.5 ± 8.19	0.025	60.5 ± 7.77	60.0 ± 6.94	0.068	63.3 ± 5.53	63.0 ± 5.05	0.057	**0.888**
Fat mass [kg]	12.9 ± 4.36	13.0 ± 4.53	0.022	12.0 ± 4.23	10.2 ± 4.17^**^	0.429	15.0 ± 4.60	12.5 ± 4.20^***^	0.568	**< 0.001**
Fat free mass [%]	83.5 ± 3.88	83.3 ± 4.05	0.050	83.7 ± 3.63	85.9 ± 3.83^**^	0.590	81.0 ± 4.78	83.6 ± 4.80^***^	0.543	**0.001**
Fat mass [%]	16.5 ± 3.88	16.7 ± 4.05	0.050	16.3 ± 3.63	14.1 ± 3.83^**^	0.590	19.0 ± 4.78	16.4 ± 4.80^***^	0.543	**0.001**
REE [kcal]	2,326 ± 285.7	2,439 ± 425.2	0.312	2,340 ± 255.1	2,329 ± 157.6	0.052	2,356 ± 259.9	2,307 ± 223.8	0.202	0.621
RER (resting)	0.804 ± 0.060	0.850 ± 0.086	0.620	0.803 ± 0.113	0.778 ± 0.065	0.271	0.809 ± 0.117	0.751 ± 0.091	0.553	0.091
**Submaximal cycle test**										
RER (activity)	0.814 ± 0.035	0.819 ± 0.038	0.137	0.806 ± 0.057	0.800 ± 0.036	0.126	0.808 ± 0.032	0.730 ± 0.043^**^	1.501	**0.009**
Lactate (resting) [mmol/l]	0.662 ± 0.125	0.832 ± 0.195^*^	1.038	0.738 ± 0.167	0.678 ± 0.147	0.381	0.647 ± 0.098	0.646 ± 0.111	0.010	**0.008**
Lactate (activity) [mmol/l]	1.39 ± 0.416	1.35 ± 0.540	0.083	1.54 ± 0.674	1.31 ± 0.443	0.403	1.56 ± 0.754	1.00 ± 0.331	0.962	0.212
Glucose (resting) [mg/dl]	88.1 ± 4.98	86.5 ± 7.02	0.263	88.7 ± 6.46	79.6 ± 10.6^*^	1.037	81.3 ± 10.8	78.0 ± 10.2	0.314	0.286
Glucose (activity) [mg/dl]	82.9 ± 5.77	77.9 ± 6.24	0.832	85.1 ± 11.4	79.9 ± 8.56	0.516	79.9 ± 6.47	77.2 ± 11.2	0.295	0.814
**Incremental cycle test**										
RER (exhaustion)	1.05 ± 0.060	1.05 ± 0.064	0.000	1.07 ± 0.063	1.09 ± 0.058	0.330	1.05 ± 0.055	0.966 ± 0.024^*^	1.980	**0.026**
Lactate (exhaustion) [mmol/l]	7.69 ± 2.03	8.81 ± 3.20	0.418	9.57 ± 2.29	9.73 ± 1.94	0.075	7.67 ± 1.22	6.34 ± 1.90	0.833	0.155
Glucose (exhaustion) [mg/dl]	84.8 ± 7.71	82.9 ± 12.1	0.187	88.2 ± 11.7	86.1 ± 13.9	0.163	88.4 ± 14.6	86.5 ± 18.0	0.116	1.000
Time to Exhaustion [min]	27.9 ± 3.43	29.3 ± 4.46	0.352	28.4 ± 4.49	29.7 ± 5.75	0.252	29.5 ± 5.92	27.7 ± 5.87^*^	0.305	**0.002**

*Data represent mean ± SD. BMI, body mass index; REE, resting energy expenditure; RER, respiratory exchange ratio; d_cohen_, effect size for comparison between baseline and final examination; P value ANOVA, Differences between groups with one-way ANOVA*.

* *p < 0.05*;

** *p < 0.01*;

*** *p < 0.001 within the group from baseline to final examination. Bold numbers represent statistical significance of the efficacy endpoints*.

Based on the results of the BIA measurements, the LGI-G (−1.83 ± 1.19 kg) and HFLC-G (−2.57 ± 1.21 kg) exhibit a statistically significantly greater loss in fat mass (LGI-G vs. HGI-G: *p* = 0.007, *d* = 1.508; HFLC-G vs. HGI-G: *p* < 0.001, *d* = 2.047) than the HGI-G (0.134 ± 0.473 kg). Similarly, the changes in the percentage fat mass in the LGI-G (−2.14 ± 1.58%) and HFLC-G (−2.62 ± 1.12%) were statistically significantly different (LGI-G vs. HGI-G: *p* = 0.009, d = 1.359; HFLC-G vs. HGI-G: *p* = 0.002, *d* = 1.842) from the changes in the HGI-G (0.178 ± 1.83%). Due to favorable changes in body fat, the increase in percentage fat free mass was also statistically significantly higher (LGI-G vs. HGI-G: *p* = 0.009, *d* = 1.359; HFLC-G vs. HGI-G: *p* = 0.002, *d* = 1.842) in the LGI-G (1.22 ± 0.974%) and HFLC-G (1.60 ± 0.656%) compared with the HGI-G (−0.167 ± 1.08%). No statistically significant differences were observed when comparing the changes in fat mass or fat free mass between LGI-G and HFLC-G.

### Metabolic Outcomes

#### Resting Conditions

The REE did not change to the level of statistical significance or the MID during the intervention period in any of the groups. In addition, there were no significant differences between the groups. Under resting conditions, the RER increased in the HGI-G by 0.047 ± 0.076 (*p* = 0.117). In the LGI-G, the RER at rest changed by −0.026 ± 0.108 (*p* = 0.475). Data of the HFLC-G revealed a non-significant decrease in RER by −0.058 ± 0.108 (*p* = 0.146). When comparing the differences in resting RER values between the LGI-G and HFLC-G, the effect was small (*d* = 0.299). The differences between the HGI-G and the LGI-G (*d* = 0.765) and the HGI-G and the HFLC-G (*d* = 1.123) were meaningful.

#### Submaximal Cycle Test

In accordance with resting conditions, the RER during the submaximal cycle test decreased in the HFLC-G from baseline to post-intervention (−0.078 ± 0.046, *p* = 0.001). In contrast, the HGI-G and LGI-G had no statistically significant changes in the RER values (Table 2). As a consequence, the changes in the RER values in the HFLC-G differed statistically significantly from the HGI-G and LGI-G as confirmed by the *post-hoc* analysis (HFLC-G vs. HGI-G: *p* = 0.014, *d* = 0.775 and HFLC-G vs. LGI-G: *p* = 0.030, d = 0.557).

After 4 weeks, the lactate concentrations at rest remained stable in the HFLC-G (−0.001 ± 0.097 mmol/l). The lactate concentrations under resting conditions decreased slightly in the LGI-G (−0.060 ± 0.206 mmol/l) and increased statistically significantly in the HGI-G (0.170 ± 0.206 mmol/l, *p* = 0.038), resulting in a significant difference between these two groups (*p* = 0.008, *d* = 1.365). The differences between the HGI-G and the HFLC-G (*d* = 1.064) were also meaningful, but not statistically significant (*p* = 0.065). After 4 weeks of intervention, the lactate concentrations during activity decreased in the HFLC-G (−0.553 ± 0.783 mmol/l, *p* = 0.067) by more than half of the SD in contrast to the LGI-G (−0.226 ± 0.547 mmol/l, *p* = 0.224) and the HGI-G (−0.041 ± 0.449 mmol/l, *p* = 0.976). Differences between groups were not statistically significant. Except for glucose concentrations under resting conditions in the LGI-G (−9.10 ± 11.3 mg/dl, *p* = 0.031), no statistical or MID relevant changes of the glucose concentrations were observed in any group in the submaximal cycle test.

#### Incremental Cycle Test

**Figure 3:**
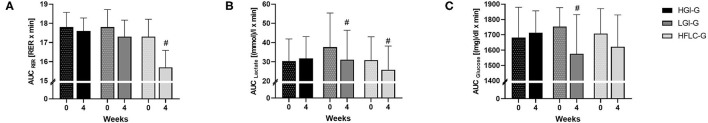
Column diagram for group differences in area under curve (AUC). **(A)** Changes in respiratory exchange ratio (RER) values, **(B)** changes in lactate concentrations, and **(C)** changes in glucose concentrations during the first 21 min of the incremental cycle test. Data shown as mean ± SD. ^#^*p* < 0.05 paired sample *t*-test for changes compared to baseline.

As shown in Figure 3A, the AUC for the RER during the incremental cycle test decreased statistically significantly (−1.64 ± 0.700 RER × minutes; *p* < 0.001) in the HFLC-G. Furthermore, *post-hoc* analysis of the ANOVA revealed statistically significant differences between the HFLC-G and the HGI-G (*p* = 0.014, *d* = 1.485) and the HFLC-G and the LGI-G (*p* = 0.044, *d* = 1.351). After 4 weeks, participants of the HFLC-G had statistically significantly (*p* = 0.014) lower RER values at exhaustion compared to baseline. In the HGI-G RER, values at maximum effort were similar at post-intervention compared to baseline. The same results could be observed in the LGI-G (Table 2). As a result of changes in RER values in the HFLC-G (−0.080 ± 0.077), the ANOVA revealed statistically significant differences between groups (*p* = 0.026). *Post-hoc* analysis showed that a HFLC diet exhibits a significantly lower RER at exhaustion than the LGI diet (*p* = 0.028, *d* = 1.351). Differences between the HGI-G and the HFLC-G were meaningful (*d* = 0.974), but did not reach the level of significance (*p* = 0.128).

Participants of the LGI-G [−6.56 ± 6.65 (mmol/l) × min; *p* = 0.012] and the HFLC-G [−5.00 ± 5.71 (mmol/l) × min; *p* = 0.030] had a statistically significant decrease in AUC for lactate concentrations during the incremental cycle test (Figure 3B). Lactate concentrations at exhaustion had not statistically significantly changed in any group. However, lactate concentrations at exhaustion increased in the HGI-G (1.12 ± 2.23 mmol/l) and decreased in the HFLC-G (−1.14 ± 2.10 mmol/l) by more than half of the SD, resulting in meaningful differences (*d* = 1.057) between the HGI-G and the HFLC-G. Nevertheless, the changes in lactate concentration at exhaustion did not differ significantly between groups in contrast to the time to exhaustion (TTE) (Table 2). TTE increased in the LGI-G (1.30 ± 1.97 min; *p* = 0.067) and HGI-G (1.40 ± 1.92 min; *p* = 0.060), whereas participants of the HFLC-G (−1.79 ± 2.00 min; *p* = 0.027) had a statistically significantly lower TTE after 4 weeks of intervention, resulting in statistically significant differences compared to the LGI-G (*p* = 0.006, *d* = 1.557) and HGI-G (*p* = 0.006, *d* = 1.629).

Blood glucose concentrations during the incremental test decreased in the LGI-G [−178.4 ± 207.0 (mg/dl) × min] and the HFLC-G [−86.9 ± 191.1 (mg/dl) × min], but reached only in the LGI-G the level of statistical significance (*p* = 0.023) (Figure 3C). Glucose concentrations at exhaustion did not change between baseline and post-intervention in any group. Furthermore, no group differences could be detected for glucose concentrations during the incremental test or at exhaustion.

### Visual Analog Scale

**Figure 4:**
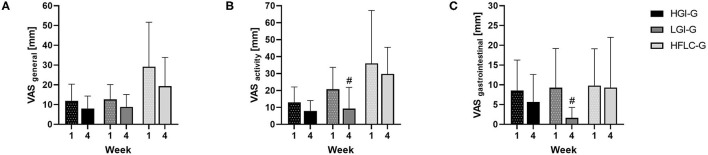
Changes in visual analog scale (VAS) Scores. **(A)** General, **(B)** during physical activity, **(C)** gastrointestinal comfort. Data shown as mean ± SD at week 1 and week 4. ^#^*p* < 0.05 Wilcoxon signed-rank test for changes compared to baseline.

Changes in the VAS Score are shown in Figure 4. VAS Scores revealed changes in general (HGI-G: −3.9 ± 10.7 mm, Md = −5.1 mm; LGI-G: −3.8 ± 7.9 mm, Md = −2.6 mm; HFLC-G: −9.9 ± 19.6 mm, Md = −3.6 mm), during physical activity (HGI-G: −5.0 ± 9.8 mm, Md = −4.6 mm; LGI-G: −11.5 ± 9.3 mm; Md = −11.2 mm; HFLC-G: −6.3 ± 20.0 mm, Md = 1.2 mm), and in gastrointestinal conditions (HGI-G: −2.9 ± 4.0 mm, Md = −1.9 mm; LGI-G: −7.6 ± 8.5 mm, Md = −7.4 mm; HFLC-G: −0.1 ± 7.3 mm, Md = −1.9 mm). Only in LGI-G could statistically significant improvements from week 1 to 4 be detected for VAS subscale ‘activity' (*p* = 0.008, *d* = 0.899) and VAS subscale ‘gastrointestinal' (*p* = 0.043, *d* = 1.052). For all other analyses, no statistical or meaningful differences between week 1 and 4 could be detected in the respective group. Except for the VAS subscale ‘activity' (*p* = 0.012), the Kruskal–Wallis test showed no statistical group differences for the different VAS values. *Post-hoc* analysis revealed a statistically significant difference between the LGI-G and HFLC-G (*p* = 0.005, *d* = 0.340) and the HGI-G and HFLC-G (*p* = 0.021, *d* = 0.083).

## Discussion

The main purpose of the present investigation was to examine the effect of nutrition strategies varying in amount and type of carbohydrates on metabolic processes under resting conditions and different exercise scenarios. Compared to baseline levels, lactate concentrations under resting conditions and in submaximal test settings decreased in the group consuming low glycemic carbohydrates in a period of 4 weeks. During the incremental test, changes in lactate concentration were statistically significant and metabolically relevant. Although the fat oxidation was not measured directly in the current investigation, evidence suggests that there is a strong inverse relationship between plasma concentrations of lactate, free fatty acids, and β-oxidation during exercise (31). As a potential consequence, the alterations in lactate concentrations might be indicative for an influence of a LGI diet on fat metabolism. Consuming <50 g carbohydrates per day in the HFLC-G over 4 weeks resulted in a practically relevant and statistically significant decrease of RER values both under resting conditions and submaximal exercises, indicating an increased fat oxidation. These finding were supported by the changes in lactate concentrations during exercise. In the current investigation, lactate concentrations decreased in the HFLC-G during the submaximal and the incremental cycle test. As a potential result of low baseline data, lactate concentrations under pre-exercise conditions remained unchanged in the HFLC-G.

In the last decades, several studies have investigated the metabolic adaptations by long-term (≥2 weeks) HFLC diets. Various investigations (≥2 weeks) have identified an increased utilization of fats measured by decreased RER and lactate values and an increased fat oxidation during resting and submaximal exercise conditions (14, 32–36). In contrast, the carbohydrate-rich control diets were associated with the opposite effect (32, 33, 35). The reciprocal relationship between fat and carbohydrate oxidation in muscle at rest and during different exercise scenarios might be explained by higher plasma concentrations of free fatty acids, decreased insulin concentrations, and improved fat transportation via fatty acid translocase FAT/CD36 protein across the cell membrane resulting from an increased fat intake (2, 3). Furthermore, major carbohydrate metabolizing enzymes (glycogen phosphorylase, phosphofructokinase, and pyruvate dehydrogenase) are less activated, while the activity of hormone-sensitive lipase and adipose triacylglycerol lipase has been shown to be increased. Carbohydrate-induced high plasma insulin concentrations caused the opposite effects (2, 3).

It has to be mentioned that in most studies, the carbohydrate-rich controls have been defined by the amount but not the GI of the ingested carbohydrates. The consumption of low glycemic carbohydrates is characterized by reduced postprandial glucose concentrations, which stimulates less insulin release. Consequently, the associated effects in the carbohydrate and fat metabolism, such as reduced lactate concentrations, decreased RER values, and increased use of free fatty acids, could be identified despite a high amount of carbohydrates (15–17, 19). However, there are controversial results whether low glycemic vs. high glycemic meals prior to exercise improved fat oxidation and performance during exercise (37).

To our best knowledge, there is little evidence coming from studies that have focused on longer-term low GI diets. In a study by Hamzah et al. the effect of the GI of high carbohydrate diets on energy metabolism and running capacity have been investigated (23). The authors concluded that the GI had no influence on rates of fat oxidation. Taking metabolic adaptations to HFLC diets under consideration, 5 days might be insufficient for a LGI diet to have an impact on the metabolic response (9, 10). A long-term effect has only been investigated in a study by Durkalec-Michalski et al. In contrast to our findings, the LGI diet over 3 weeks resulted in a slight downregulation of fat oxidation during exercise (38). However, in the study by Durkalec-Michalski et al., only rates of fat oxidation were measured while in the current investigation, changes in lactate concentrations were measured to assess the metabolic impact of the respective diets. Furthermore, the decrease in RER values was not statistically significant and did not reach the MID in the present study.

The LGI intervention seemed to have a smaller impact on metabolic adaptations than the HFLC diet. The up-regulating signals of fat oxidation are low insulin concentration and increased concentrations in plasma free fatty acids (2, 3). A direct comparison between four meals, each different in the amount and GI of the ingested carbohydrates, has shown that both high fat groups were associated with the highest postprandial free fatty acid and lowest insulin concentrations. The lowest free fatty acid concentrations were in the group consuming a low glycemic carbohydrate-rich meal. Furthermore, postprandial insulin response was lower in the high carbohydrate low GI group compared to the high carbohydrate high GI group (39). Consequently, the abovementioned adaptation processes might be less in a high carbohydrate low glycemic diet compared to a HFLC diet due to the different impact on postprandial free fatty acid and insulin concentrations.

The nutritional impact on fat metabolism might also be reflected by the circulating glucose concentrations. Fasting glucose plasma concentrations dropped in the LGI-G to a significant and MID relevant extent. Changes in the HFLC-G seemed to be less pronounced, potentially as a consequence of relatively low baseline values compared to the other groups. During the post-intervention, incremental test glucose concentrations are lower at the same exercise intensity as in the unconditioned (pre-values) state in both LGI-G and HFLC-G. This is probably related to a stimulation of fat oxidation under resting conditions and during exercise (40).

The results of the HGI-G seemed to be controversial. The increased RER at rest in the HGI-G indicates an elevated metabolization of carbohydrates under resting conditions. In addition, the lactate concentration increase was clinically relevant under pre-exercise condition. Despite increased lactate concentrations during the incremental test, it seems that there is an improved fat metabolism -decreased glucose and lactate values- in the submaximal cycle test. It had previously been described that carbohydrates prior to exercise appear to be beneficial to performance (1). Hence, the slightly decreased carbohydrate metabolism in the submaximal test might be partly explained by the increased lactate threshold over the time as a possible adaptation in response to enhanced performance. As a result, at post-intervention, the participants performed the test closer to their lactate threshold compared to baseline.

The current investigation also observed an improvement in body composition due to a decrease in fat mass following the 4-week LGI or HFLC diet on the level of significance and MID. It is not assumed that the present results can be attributed to the differences in energy intake between groups. Despite the significant difference in proportions of nutrients, the mean energy intake was equivalent between groups with an energy add-on of 100 kcal in the HFLC-G. According to the findings of Hall et al., the additional daily energy intake needs to amount to about 215 kcal (900 kJ) to induce weight gain (41).

There is evidence that athletes can improve their body composition by a high fat (in particular ketogenic) diet (42–44). Low carbohydrate diets compared with control diets have been suggested to be relatively more effective in body weight management. However, the benefits of a low carbohydrate diet can be rather attributed to the relatively high protein content, but not the relatively lower carbohydrate content (45, 46). In a recent study with athletes, different approaches (high vs. low fat) but similar protein intakes resulted in a similar change of body composition (mean loss in body fat was 1.4 kg) (32). These are in accordance with a meta-analysis examining the impact of different diet types in obese or overweight people (47). Data from the meta-analyses of the Cochrane Database of Systematic Reviews suggest that a low glycemic diet without energy restriction results in a significantly greater decreased fat mass and an increased fat free mass compared with a high glycemic or even high fat and energy restricted diet (48). Although low glycemic diets seem to promote weight loss and metabolic improvements in obese and overweight adults (48), research about the impact of the GI on body composition in endurance athletes is limited. A recent study by Durkalec-Michalski et al. has shown that consuming a low glycemic diet led to a change in body composition. In particular, a statistically significant reduction in body mass (49). Physiologically, the significant changes in body composition in the present investigation might be explained by changes in fat oxidation and a more balanced carbohydrate metabolism as a potential consequence of the altered amount and quality of ingested carbohydrates.

Despite an improvement in fat metabolism and body composition, there is a growing body of evidence that these changes induced by ketogenic or non-ketogenic HFLC diets are not in association with improved endurance performance, aerobic capacity and peak performance in particular (9, 32, 50, 51), due to an impaired carbohydrate provision during higher intensities (2). This assumption is supported by the changes in time to exhaustion in the present investigation.

Furthermore, HFLC diets seem to be impractical and accompanied by side effects that include fatigue, headaches, poor concentration, lethargy, gastrointestinal discomfort, nausea, and unintentional weight loss. One reason might be an insufficient proliferation of essential micronutrients and fibers and glycogen depletion which might be a cause of impaired concentration and hence the neuromuscular connection (9, 52).

The values of the VAS scores of all categories decreased in all groups, indicating that the participants got familiar with the respective dietary concepts. In general, none of the groups experienced clinically relevant elevated VAS scores. Mild symptoms can be defined by a score of 5 to 45 mm on the VAS (53). This might be explained by the fact that endurance subjects tolerate the effects of a high-fat diet better than untrained individuals during exercise (54). In addition, according to the nutritional protocols, an impaired delivery of minerals in the HFLC group was not expected.

However, only the LGI-G and HGI-G have shown an improvement in VAS scores of the subscale activity and gastrointestinal comfort on a statistical or MID level with a superior effect in the LGI-G. During the last week of intervention, the HFLC-G had statistically significant higher VAS scores in the subscale ‘activity' compared with the LGI-G or HGI-G. These results might be associated with impaired training sessions in the HFLC-G since higher intensity levels could not be reached without the provision of carbohydrates (2). Furthermore, the advantage of LGI diet over HFLC and HGI diets might be in the choice of carbohydrates. A LGI diet is predominantly characterized by high-fiber and plant-based foods. This has shown to be associated with reduced fatigue, a strengthened immune system, and an improved ability to regenerate through the increased supply of micronutrients, essential fatty acids and amino acids, and low postprandial glucose concentrations (55). Moreover, controlled clinical trials demonstrated that low glycemic foods have a positive impact on digestive conditions, such as gastroesophageal reflux disease or the irritable bowel syndrome, due to high fiber content (56, 57).

It can be assumed that the present results can be attributed to the implementation of nutritional patterns. According to the analysis of the nutritional protocols, the participants' dietary intake reflected the specified intake of carbohydrates and fats in the respective group. While the HGI-G had a higher percent and total carbohydrate intake, the LGI-G showed a higher carbohydrate intake on a g-per-kg-body-weight basis. The current guidelines for endurance athletes during training on the competition level are 6–10 g carbohydrates per kg body weight and day. These recommendations do not address the GI of the ingested carbohydrates (58). The participants of the current investigation were non-elite athletes with a training workload of 3–5 sessions per week. In both groups, the carbohydrate intake seems to be sufficient since recommendations are 5–7 g carbohydrates per kg bodyweight and day for general training needs (58). Nevertheless, increasing the carbohydrate intake to 6–10 g carbohydrates per kg body weight and day would be an interesting approach in future studies with high trained endurance athletes. The carbohydrate upper limit of 50 g per day in the HFLC-G was based on the current focus of carbohydrate-restricted diets (9). Furthermore, the mean fat intake was 65%. It is postulated that the proportion of fat metabolism can be increased if fat supplies 50–70% of the total energy (2).

This trial has some limitations. It has to be mentioned that the changes in fat and carbohydrate oxidation were not measured directly but extrapolated from the lactate diagnostics. However, it is reported that measuring blood lactate is an effective way to estimate the rates of fat and carbohydrate oxidation (59). Furthermore, using the values of the spiroergometry to confirm the results from the lactate diagnostic during the incremental test has to be taken with caution since values for VO_2_ are overestimated by a step compared to a ramp incremental test (60). When taking the impact of the nutritional concepts into account, limitations of the self-reported protocols might entail an over or underreporting of the consumed foods (61). Moreover, recommendations for the macronutrient intake based on the body weight seems to be more accurate than percentage values to determine nutritional guidelines for endurance athletes. Future studies with a larger sample size should include different sex groups and pre-exercise nutritional conditions to state practical use of high fat vs. high carbohydrate diets. Furthermore, the analysis of the muscle glycogen would be helpful for a better interpretation of the energy supply (9). Ultrasonic assessment can be used to quantify glycogen content in the skeletal muscle (62).

## Conclusion

In conclusion, the effect of the LGI diet was a decrease in lactate concentrations under resting and submaximal exercise conditions, while HFLC diet resulted additionally in decreased RER values. However, these lower adaptations in the LGI-G seem to be beneficial in terms of an enhanced metabolic flexibility, since an increased carbohydrate metabolism was unaffected during higher intensities, while the utilization of fats was facilitated during submaximal exercise due to decreased plasma lactate concentrations. Despite the positive impact on the fat oxidation and body composition, following a HFLC diet might have a negative effect on exercise performance due to the lack of carbohydrate provision at higher intensity levels. In addition, there might be negative long-term health consequences due to the high fat content and decreased intake of essential micronutrients. The HGI-G changes in metabolism might impair the ability to effectively use fats and carbohydrates during different exercise intensities. Taking these findings together, the implementation of a LGI diet leads to a more flexible fat and carbohydrate metabolism after 4 weeks of intervention in contrast to a HFLC or HGI diet, which might be of advantage, particularly during strenuous endurance exercise.

## Author's Note

After the study was finished, DZ started as a researcher in the Collagen Research Institute, Kiel.

## Data Availability Statement

The raw data supporting the conclusions of this article will be made available by the authors, without undue reservation.

## Ethics Statement

The study was conducted according to the guidelines of the Declaration of Helsinki and approved by the Independent Ethics Committee of the University of Freiburg (protocol code: 136-16 and date of approval: 2017/10/05). The patients/participants provided their written informed consent to participate in this study.

## Author Contributions

DZ, HF, AG, and DK designed the study. DZ, HF, and DK were responsible for data acquisition and performed the analysis. All authors read and approved the final version of the manuscript.

## Conflict of Interest

The authors declare that the research was conducted in the absence of any commercial or financial relationships that could be construed as a potential conflict of interest.

## Publisher's Note

All claims expressed in this article are solely those of the authors and do not necessarily represent those of their affiliated organizations, or those of the publisher, the editors and the reviewers. Any product that may be evaluated in this article, or claim that may be made by its manufacturer, is not guaranteed or endorsed by the publisher.
