# Supplementary material for: Effect of a High Fat Diet vs. High Carbohydrate Diets With Different Glycemic Indices on Metabolic Parameters in Male Endurance Athletes: A Pilot Trial
Source: Front Nutr. 2022 Apr 11;9:802374. doi: 10.3389/fnut.2022.802374 (PMC9037589; doi:10.3389/fnut.2022.802374)
Supplement: Supplementary file 1 [file Data_Sheet_1.docx]

Supplementary Material

Table S1: Foods consumed in the respective group during the 4-week intervention

|  | **HGI-G** | **LGI-G** | **HFLC-G** |
| --- | --- | --- | --- |
| General information   - Carbohydrates - Fats^1^ - Proteins | ≥ 65 % (GI > 70)  ≤ 15 %  15 – 20% | ≥ 65 % (GI < 50)  ≤ 15 %  15 – 20% | ≤ 50 g per day  ≥ 65 %  15 – 20% |
| Meat, fish, eggs | No limits, but in small amounts, rather low-fat variants (e.g., steak, salmon, chicken breast, cold cuts, eggs) | No limits, but in small amounts, rather low-fat variants (e.g., steak, salmon, chicken breast, cold cuts, eggs) | No limits except for breaded products as fish sticks (e.g., steak, salmon, chicken breast, cold cuts, eggs) |
| Vegetables | Potato (products)  All others only as side dish, but not main carbohydrate serving | No limits except for potatoes | Low starch vegetables as cabbages, cucumber, salat, tomatoes, peppers; No high-starch vegetables as (sweet) potatoes, legumes or maize |
| Fruits | Dates (dried), sugared fruits  Bananas and grapes allowed | No limits except for dates, bananas, grapes and sugared fruits | ≤ 200 g, fruits low in sugar as berries, papayas and watermelon |
| Dairy products | Sugared dairy products as fruit yoghurts, low fat variants should be preferred, mature cheese with a high fat content should be eaten occasionally  Rice drink as a high GI alternative for milk | Low fat products as low-fat curd, low fat yoghurt, cottage cheese, buttermilk; mature cheese in small amounts, no sugared or flavored industrial dairy products | No fresh dairy products (e.g., milk, some low fat and fruit yoghurts) due to the high content of carbohydrates; butter mature cheese, whole yoghurt and cream cheese with a low content of carbohydrates are allowed |
| Grain products^2^ | White flour products as white bread, toast but also white rice, millet, couscous, bulgur, pasta, cereals and potatoes  No whole grain (bread, rice, pasta) and pseudocereals | Whole grain products as whole-grain bread, rice and pasta  Pseudocereals  Oats | Due to the high content of carbohydrates not allowed; low-carb breads made of nuts and coconut or almond flour can replace conventional bread |

Table S1: Foods consumed in the respective group during the 4-week intervention (continued)

|  | **HGI-G** | **LGI-G** | **HFLC-G** | |
| --- | --- | --- | --- | --- |
| Nuts and seeds | No limits, but in small amounts (source of fat) | No limits, but in small amounts (source of fat) | No limits except for chestnuts, cashews and pumpkin seeds | |
| Beverages | No limits for non-alcoholic drinks, alcoholic drinks should be limited and strictly avoided before examinations | Coffee, unsweetened teas, water, sugar-free lemonades and juices on the basis of the allowed fruits; no alcoholic drinks, lemonades and sugared juices | Coffee, unsweetened teas and water; no alcoholic drinks, lemonades and juices | |
| Sweeteners, sweets and snacks | Low fat snacks as gummi bears, smarties, sorbets, rice cracker, low fat crisps and sports bars, sugar | Nuts, fruits (except for bananas, grapes and dates), dark chocolate (≥ 70 % cacao), sugar-free marmalade, agave syrup, cinnamon, sugar substitutes as Stevia and Xylitol are allowed | Nuts, berries, dark chocolate (≥ 70 % cacao), cinnamon, sugar substitutes as Stevia and Xylitol are allowed | |
| ^1^ Predominantly plant-based (65 %), non-processed fats as oils, fish, nuts and seeds  ^2^ cooked *al dente* in the LGI-G to keep the GI as low as possible  The glycemic index of the foods was based on Foster-Powell et al. (2002) (see Reference 20) | | | |  |

Table S2: Meal plan examples for a high carbohydrate high glycemic diet (HGI-G)

| Breakfast | Smoothie/Muesli based on 300 g:   - Sweetened yoghurt (1.5% fat) - Rice milk   Sweetened with 30g syrup or sugar  Mixed with   - 1 banana, grapes, raisins or dried dates - 60 g cornflakes, breakfast cereals, instant muesli, puffed rice, puffed amaranth   In addition: coffee with milk and sugar or tea | Energy: 650 kcal  Protein: 21 g  Carbohydrates: 124 g  Fat: 6 g |
| --- | --- | --- |
| Breakfast | 4 pancakes with the following topping   - 30 g maple syrup, sugar - 30 g jam - 1 banana or 50 g dried dates, raisins   60 g cottage cheese/cream cheese/curd (low-fat) | Energy: 860 kcal  Protein: 28 g  Carbohydrates: 156 g  Fat: 12 g |
| Breakfast or dinner | 1-2 slices of white bread, rice cracker, toast or rusks with   - Low-fat turkey or chicken breast - Low-fat cream cheese, cottage cheese or low-fat curd - Jam - Maple or sugar beet syrup   1 banana, grapes, raisins or dried fruit  In addition: coffee sweetened with milk or tea or fruit juice containing sugar | Energy: 600 kcal  Protein: 56 g  Carbohydrates: 61 g  Fat: 11 g |
| Lunch or dinner | Chili con carne made from 100 g minced beef, 1/2 onion, 200 g tomato paste, 50 g ketchup, 150 g kidney beans, spices as required  70 g baguette or pretzel | Energy: 760 kcal  Protein: 43 g  Carbohydrates: 92 g  Fat: 25 g |
| Lunch or dinner | 80 g white or glutinous rice, 100 g pasta or 350 g potatoes, mashed potatoes or 200 g fries or gnocchi with 100 g fish, meat or seafood low in fat (e.g., chicken breast, beef, trout) and 1 small salat (ca. 40 g) | Energy: 715 kcal  Protein: 25 g  Carbohydrates: 145 g  Fat: 3 g |
| Snacks | 100 g dried dates or sugared fruits + 200 g sweetened yoghurt  100 g dried dates or sugared fruits + 30 g nuts  100 - 150 g jelly beans, toffee | Energy: 400 kcal  Protein: 10 g  Carbohydrates: 65 g  Fat: 15 g |

Table S3: Meal plan examples for a high carbohydrate low glycemic diet (LGI-G)

| Breakfast | Smoothie/Muesli based on 300 g:   - Sweetened yoghurt (1.5% fat) - Milk (1.5% fat) - Soy milk - Curd (low fat)   Mixed with:   - 200 g fruits (e.g., berries, apples, pears) - 100 g oats - 30 g agave sirup   In addition: coffee with milk or tea | Energy: 700 kcal  Protein: 25 g  Carbohydrates: 120 g  Fat: 11 g |
| --- | --- | --- |
| Breakfast | Omelet made from 2 eggs, 1 tomato, 150g mushrooms, ½ bell pepper, 5g butter, plus 2 slices of wholegrain bread  In addition: 1-2 portions of fruits, coffee with milk or tea | Energy: 750 kcal  Protein: 37 g  Carbohydrates: 81 g  Fat: 27 g |
| Breakfast or dinner | 1-2 slices of wholegrain bread with:   - Low-fat turkey or chicken breast - Low-fat cream cheese, cottage cheese or low-fat curd - Tomato paste - Mozzarella (light) - Egg - Sugar-free jam - Agave Syrup - Hummus   In addition: Vegetables, 1-2 portions of fruit and coffee with milk or tea | Energy: 600 kcal  Protein: 50 g  Carbohydrates: 70 g  Fat: 6 g |
| Lunch or dinner | 200 g vegetables cooked or raw (as a salad), 80 g brown rice, buckwheat, quinoa (raw), 100 g legumes with 100 g fish, meat or seafood low in fat (e.g., chicken breast, beef, trout) and tomato sauce, crème fraiche (10% fat) | Energy: 600 kcal  Protein: 30 g  Carbohydrates: 11 g  Fat: 17 g |
| Lunch or dinner | Gazpacho made from 170 g strained tomatoes, 170 g cucumber, 170 g belly pepper, 2 tbsp balsamic vinegar, 1 tbsp olive oil, spices as required  1 slice whole grain bread | Energy: 320 kcal  Protein: 10 g  Carbohydrates: 50 g  Fat: 8 g |
| Snack | 100 g fruits + 200 g unsweetened yoghurt  100 g fruits + 30 g nuts | Energy: 400 kcal  Protein: 10 g  Carbohydrates: 30 g  Fat: 15 g |

Table S4: Meal plan examples for a high fat low carbohydrate diet (HFLC-G)

| Breakfast | Muesli based on 300 g:   - Greek yoghurt (10% fat) - Curd (40% fat) - Coconut milk   Mixed with max. 200g low-sugar fruit (berries, papaya, watermelon) and approx. 50 g nuts or seeds  In addition: coffee with cream or tea | Energy: 670 kcal  Protein: 21 g  Carbohydrates: 30 g  Fat: 47 g |
| --- | --- | --- |
| Breakfast | Omelet made from 3 eggs, 1 tomato, 150g mushrooms, 50g avocado and 30g parmesan, 1 tbsp olive oil | Energy: 700 kcal  Protein: 41 g  Carbohydrates: 14 g  Fat: 55 g |
| Breakfast or dinner | 2 slices of low carb bread with:   - (Pea)nut butter - Sausage (salami, liver sausage, mortadella, bacon) - Cheese 45% (e.g., Emmental, Appenzell, Gouda) - Double cream cheese - Butter - Hummus - Avocado (also as guacamole) - Smoked salmon - Egg   Vegetables as required, 1-2 portions of fruit (max. 200 g) and coffee with cream or tea | Energy: 700 kcal  Protein: 32 g  Carbohydrates: 20 g  Fat: 58 g |
| Lunch or dinner | Chili con carne made from 150 g minced beef, 1/2 onion, 250 g tomato paste, 50 g kidney beans, 1 tbsp oil, spices as required | Energy: 570 kcal  Protein: 38 g  Carbohydrates: 14 g  Fat: 50 g |
| Lunch or dinner | Coconut soup made from 200 mL coconut milk with 150 g seafood, 100 g zucchini, 100 g belly pepper, curry and other spices as required | Energy: 650 kcal  Protein: 32 g  Carbohydrates: 10 g  Fat: 45 g |
| Lunch or dinner | 150 g fish, meat or seafood high in fat (e.g., mackerel, salmon, herring, beef) with 200 g vegetables cooked or raw (as a salad) and low-carb dressing or sauces (e.g., pesto; guacamole; vinaigrette; crème fraiche (at least 30% fat), sour cream sauce, olive oil) | Energy: 800 kcal  Protein: 50 g  Carbohydrates: 10 g  Fat: 64 g |
| Snack | 100 g low-sugar fruit (berries, papaya, watermelon) and 100 g nuts or seeds | Energy: 400 kcal  Protein: 20 g  Carbohydrates: 20 g  Fat: 50 g |
